# ScaPD: a database for human scaffold proteins

**DOI:** 10.1186/s12859-017-1806-6

**Published:** 2017-10-03

**Authors:** Xiaomei Han, Jenny Wang, Jie Wang, Sheng Liu, Jianfei Hu, Heng Zhu, Jiang Qian

**Affiliations:** 1 Department of Ophthalmology, Johns Hopkins School of Medicine, Baltimore, MD USA; 2 Department of Gastroenterology, Zhejiang Provincial People’s Hospital, Hangzhou, Zhejiang China; 3 The Horace Mann School in Bronx, Bronx, NY USA; 4 Department of Pharmacology and Molecular Sciences, Johns Hopkins School of Medicine, Baltimore, MD USA; 5 The Sidney Kimmel Comprehensive Cancer Center, Johns Hopkins School of Medicine, Baltimore, MD USA; 6 Center for High-Throughput Biology, Johns Hopkins School of Medicine, Baltimore, MD USA

**Keywords:** Scaffold protein, Signaling pathway, Database

## Abstract

**Background:**

Scaffold proteins play a critical role in an increasing number of biological signaling processes, including simple tethering mechanism, regulating selectivity in pathways, shaping cellular behaviors. While many databases document the signaling pathways, few databases are devoted to the scaffold proteins that medicate signal transduction.

**Results:**

Here, we have developed a user-friendly database, ScaPD, to describe computationally predicted, experimentally validated scaffold proteins and associated signaling pathways. It currently contains 273 scaffold proteins and 1118 associated signaling pathways. The database allows users to search, navigate and download the scaffold protein-mediated signaling networks.

**Conclusions:**

Manually curated and predicted scaffold protein data will be a foundation for further investigation of the scaffold protein in the signal transduction. With maintained up-to-date data, ScaPD (http://bioinfo.wilmer.jhu.edu/ScaPD) will be a valuable resource for understanding how individual signaling pathways are regulated.

## Background

About 10% of proteins expressed in human cells are involved in the signal transduction [1]. How can signaling proteins interact with the correct partners and avoid wrong proteins? One principle is that cells achieve well in the signal transduction networks by tethering subset proteins in space and time. More than 20 years ago, the first set of scaffold proteins were discovered, which assemble components of diverse pathways at the plasma membrane or subcellular compartments [2–6]. For example, scaffold protein Ste5 tethers multiple protein kinases in the MAP kinase cascade, such as Ste11, Ste7 and Fus3. The spatial organization achieves high efficacy information transfer on cellular information flow.

The scaffold proteins link multiple signaling proteins together to facilitate signal transduction [6, 7]. These proteins mediate a linear pathway among many partner proteins, and mediate pathway branching to multiple outputs as well [8, 9]. One central role of scaffold proteins is to coordinate feedback loops in signaling pathways, and thus to regulate the signaling response [10, 11]. They enhance signaling specificity or increase the signaling efficiency by increasing the local concentration of signaling components. Thus, the scaffold proteins play a crucial role in the signal transduction.

Although various signaling pathways are the central topics in many biological fields, researchers pay much less attention on the scaffold proteins. One possible reason is that identification of scaffold proteins is challenging, which requires multiple steps using traditional biochemical techniques, including selection of a candidate scaffold protein, testing the protein-protein interaction and assessment of the signaling pathway. The systematic study of scaffold proteins can greatly enhance the understanding of the protein regulation that occurs in eukaryotic organisms [12–14].

While many databases were constructed to collect the information of signaling pathways (e.g. KEGG, phosphonetworks, phosphoGRID) [15–17], these databases often contain little information of the scaffold proteins. We believe that a central portal specifically designed for scaffold proteins will provide a useful resource to the research community. To facilitate usage of the information of scaffold proteins, we created a scaffold protein database, ScaPD, an integrated information system for the storage and visualization of human scaffold proteins as well as the corresponding signaling pathway data.

## Results

The content of the database has two major sources. First, we performed a manual curation of the literatures to collect experimentally determined scaffold proteins. We first searched papers containing the keyword “scaffold protein” through Google Scholar and PubMed. We then manually examined the papers and collected the known scaffold proteins. In total, we collected 82 scaffold proteins. Second, we collected predicted scaffold proteins generated from a recent project, in which we developed a bioinformatics approach to predict scaffold proteins [18]. In brief, we constructed a composite network, including 55,048 protein-protein interactions and 1103 kinase-substrate relationship in human. We then identified the proteins that interact with multiple components in a signaling pathway. Based on our analysis, 212 proteins were predicted as scaffold proteins with statistical significance.

**Fig. 1:**
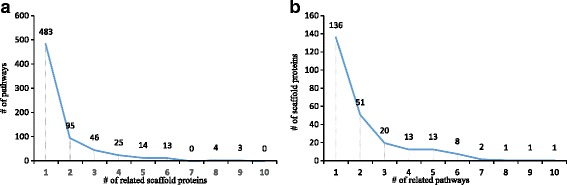
Statistical analysis of scaffold proteins and pathways. **a** Number of signaling pathways associated to scaffold proteins. **b** Number of scaffold proteins associated to pathways

In total, ScaPD collected 273 scaffold proteins and 683 distinct scaffold-mediated phosphorylation pathways. The association between scaffold proteins and signaling pathways are specific. In fact, 483 (70%) of signaling pathways are associated with only one scaffold protein (Fig. 1a), and 136(51%) of scaffold proteins are associated with one pathway (Fig. 1b).

**Fig. 2:**
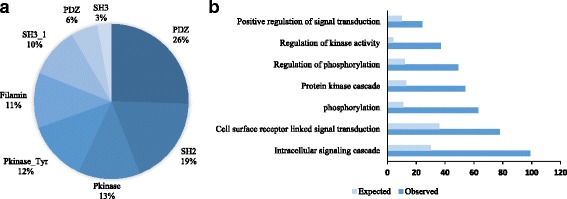
Structural and functional characterization of scaffold proteins. **a** Pfam protein domains which is greater than 20 in scaffold proteins. **b** Gene ontology analysis of scaffold proteins

The scaffold proteins often contain certain protein domains based on Pfam annotation [19]. The most prevalent domains are PDZ (26%), SH2 (19%) and Pkinase domains (13%) (Fig. 2a). The gene ontology (GO) annotation analysis indicates that 99 of the 273 scaffold proteins are associated with “intracellular signal transduction” (*p* < 1 × 10^−39^, hypergeometric distribution), and that 75 of predicted scaffold proteins with “phosphorylation” (*p* < 1 × 10^−23^, hypergeometric distribution), both over three-fold enrichment than expected group (Fig. 2b).

**Fig. 3:**
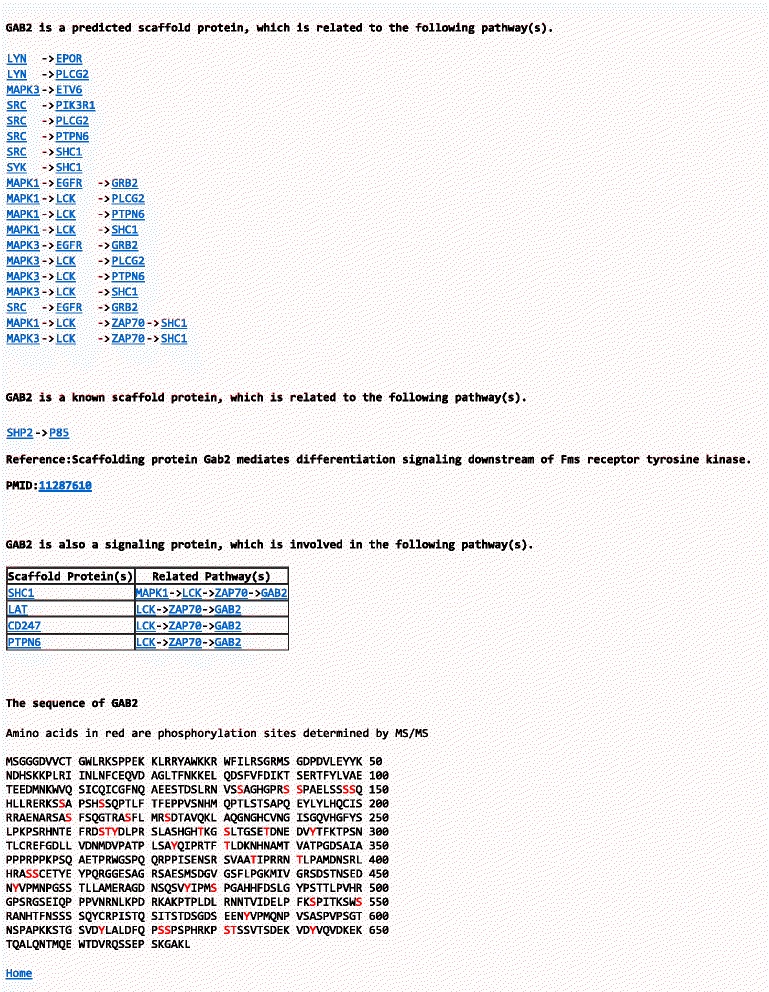
The ScaPD example for protein GAB2. The proteins is a scaffold protein and also a kinase

Users can input any human protein name, and depending on whether the protein of entry is a scaffold protein and/or signaling protein, ScaPD will return a corresponding information page for the input protein. If the protein is a scaffold protein, the page will list the associated signaling pathways. Since the scaffold proteins are likely to be regulated through phosphorylation [18], the known phosphorylation sites are highlighted in the protein sequence. If the input protein is a signaling protein, the page will list the scaffold proteins that are associated with the pathways which the input protein is involved. Note that the protein names in the return pages are all clickable so that the users can navigate through the scaffold protein-mediated signaling pathways. In addition, we also provide the reference(s) that described the scaffold protein of interest (Fig. 3).

## Discussion and Conclusion

Recent studies have revealed that the scaffold proteins play a versatile and important role in many signaling pathways. However, only a few scaffold proteins have been extensively characterized. Furthermore, no database has been developed for analyzing scaffold proteins, although many databases exist for signaling pathways. To our knowledge, ScaPD is the most comprehensive database focused on the scaffold proteins and associated signaling pathways. It holds a significant number of predicted scaffold proteins and their associated signaling pathways, which were previously completely uncharacterized. In addition, the database is more than a list of scaffold proteins. The users can search for scaffold proteins or singlaing pathways and their associated scaffold proteins. We will continuously update the scaffold proteins as new data are brought forth. Therefore, the ScaPD should provide additional information on the function of the scaffold proteins and pathways in signal transduction.
